# The different outcomes between breast-conserving surgery plus radiotherapy and mastectomy in metaplastic breast cancer: A population-based study

**DOI:** 10.1371/journal.pone.0256893

**Published:** 2021-09-02

**Authors:** Lin-Yu Xia, Wei-Yun Xu, Qing-Lin Hu

**Affiliations:** 1 Department of Thyroid and Breast Surgery, The First Affiliated Hospital of Chengdu Medical College, Chengdu, Sichuan, China; 2 Department of Breast Surgery, Mianyang Central Hospital, Mianyang, Sichuan, China; Gangnam Severance Hospital, Yonsei University College of Medicine, REPUBLIC OF KOREA

## Abstract

**Background:**

Metaplastic breast cancer (MBC) are rare. The survival outcomes of MBC patients after breast conserving surgery plus radiotherapy (BCS+RT) or mastectomy have not been established. The study aimed to compare survival outcomes of MBC patients subjected to BCS+RT or mastectomy therapeutic options.

**Methods:**

Patients who were subjected to BCS+RT or mastectomy between 2004 and 2014 were enrolled in this study through the Surveillance, Epidemiology and End Results (SEER) database. Breast cancer-specific survival (BCSS) and the overall survival (OS) of the participants were determined. Cox proportional hazard model and the Kaplan Meier method were used to determine the correlation between the two surgical methods and survival outcomes.

**Results:**

A total of 1197 patients were enrolled in this study. Among them, 439 patients were subjected to BCS+RT, while 758 patients were subjected to mastectomy. After propensity score matching (PSM), the BCS+RT and mastectomy groups consisted of 321 patients, respectively. The univariate and multivariate analysis with a 6-month landmark all indicate that patients receiving BCS+RT has higher OS than patients receiving mastectomy (HR = 0.701,95% CI = 0.496–0.990, *P* = 0.044; HR = 0.684,95% CI = 0.479–0.977, *P* = 0.037) while the BCSS was no difference between the two groups (HR = 0.739,95% CI = 0.474–1.153, *P* = 0.183; HR = 0.741,95% CI = 0.468–1.173, *P* = 0.200).

**Conclusion:**

The BCS+RT therapeutic option exhibits a higher OS in MBC patients compared to the mastectomy approach.

## Introduction

Metaplastic breast cancer (MBC) is a rare pathological type of breast cancer that is characterized by the presence of epithelial and mesenchymal components. It accounts for 1%-2% of all breast cancers [1, 2]. Compared with invasive ductal carcinoma, MBC tumors are often larger, less likely to have nodal metastasis, more likely to be hormone receptor and human epidermal growth factor receptor 2(HER 2) negative [3]. It’s more aggressive and has a poor prognosis [4–6]. MBC more commonly manifests as triple-negative disease, so endocrine therapy and targeted therapy are rarely used [7]. The effect of chemotherapy may be limited, while radiotherapy has been proved to improve the prognosis of MBC [8, 9]. Surgical treatment is still one of the important treatment methods.

Currently, surgical therapeutic options for breast cancer include breast conserving surgery and total mastectomy. Several studies have reported that BCS + RT exhibits the same survival outcomes as mastectomy [10–13]. However, given the aggressive and poor MBC prognosis, it is not certain whether a more aggressive locoregional approach is necessary. Few MBC patients are subjected to BCS when compared to the number subjected to mastectomy [2, 14]. Notably, there are no specific MBC treatment guidelines and consensus because it is a rare type of cancer. In addition, the prognosis of MBC patients after being subjected to BCS+RT and mastectomy has not been widely reported, and neither has it been established [15]. In this study, we compared the OS and BCSS of MBC patients who had been subjected to BCS+RT and mastectomy.

## Materials and methods

### Patients

This retrospective study was conducted using the SEER database published in November 2018 and contains data from 18 population-based cancer registries.

Patients diagnosed with metaplastic breast cancer from 2004 to 2014 were collected. Other inclusion criteria included: (1) female; (2) 20–79 years old; (3) T1-3N0-3M0; (4) A mastectomy or breast-conserving surgery was performed. Exclusion criteria included: (1) prophylactic mastectomy was performed; (2) patients with unknown clinical and pathological characteristics; (3) patients without radiotherapy after breast conserving surgery; (4) patients receiving neoadjuvant radiotherapy.

We collected the following clinical and pathological features: age and year of diagnosis, race, marital status, histological grade, tumor size (T stage), lymph node status, ER, PR, surgical method, postoperative chemotherapy and radiotherapy.

### Outcome measures and statistical analysis

Our main outcomes of interest was OS and BCSS, OS were calculated from the date of diagnosis to the date of death and the BCSS were calculated from the date of diagnosis to the date of death due to breast cancer.

In order to reduce the selection bias and achieve balance covariates across treatment groups, we created a matched dataset using one-to-one (1:1) PSM [16, 17]. The conditional landmark analysis was used to address a lead time bias among the propensity matched cohort [18]. With the landmark, analysis was restricted to the patients who survived to 6 months without death or loss to follow-up.

We compared the clinicopathological characteristics of the two groups of patients before and after PSM through the X^2^ test. The survival curve was plotted through the Kaplan-Meier product limit method and compared by the log rank test. A Cox proportional hazards regression model was used for the univariate and multivariate analyses of BCSS and OS. All *P* values were two-sided, and *P* < 0.05 was considered to be statistically significant. These analyses were performed using the SPSS version 20.0 software package (IBM SPSS Statistics, Chicago, IL, US).

### Ethics statement

This study obtained data from the SEER database and did not require ethical consent, because all data were fully anonymized and were publicly available.

## Results

### General characteristics of the study population and tumor

A total of 1197 patients were enrolled in the study through the SEER database. Participants were allocated into two groups based on the surgical method. They were subjected to the BCS+RT group (439, 36.68%) and the mastectomy group (758, 63.32%). Patients in the BCS+RT group showed smaller tumors, fewer lymph node metastases, higher PR negative rate, and more likely to receive chemotherapy and radiotherapy (*P*<0.05). Considering the difference between case and control groups, we used PSM to construct a matched sample consisting of 321 pairs of BCS+RT and mastectomy subjects. There was no difference between the variables of the two groups after PSM. Table 1 shows the demographic and clinicopathological characteristics of the two groups.

**Table 1:** Baseline characteristics of the study population and tumor.

Characteristics		before PSM^a^		*P*	after PSM		*P*
		BCS+RT^b^ (n,%)	Mastectomy (n,%)		BCS+RT (n,%)	Mastectomy (n,%)	
**No. of patients**		439(36.68%)	758(63.32%)		321	321	
**Year of diagnosis**	2004–2009	195(44.42%)	328(43.27%)	0.7	135(42.1%)	134(41.7%)	0.936
	2010–2014	244(55.58%)	430(56.73%)		186(57.9%)	187(58.3%)	
**Age (years)**	20–49	102(23.23%)	181(23.88%)	0.8	80(24.9%)	72(22.4%)	0.458
	50–80	337(76.77%)	577(76.12%)		241(75.1%)	249(77.6%)	
**Race**	White	336(76.54%)	563(74.27%)	0.121	256(79.8%)	262(81.6%)	0.189
	Black	79(18%)	129(17.02%)		50(15.6%)	37(11.5%)	
	Other	24(5.47%)	66(8.71%)		15(4.7%)	22(6.9%)	
**Marital status**	Married	271(61.73%)	441(58.18%)	0.228	205(63.9%)	206(64.2%)	0.934
	Not married	168(38.27%)	317(41.82%)		116(36.1%)	115(35.8%)	
**Grade**	I	16(3.64%)	18(2.37%)	0.098	5(1.6%)	7(2.2%)	0.313
	II	66(15.03%)	85(11.21%)		31(9.7%)	33(10.63%)	
	III	344(78.36%)	624(82.32%)		278(86.6%)	266(82.9%)	
	IV	13(2.96%)	31(4.22%)		7(2.2%)	15(4.7%)	
**Tumor size (cm)**	<2	183(41.69%)	150(19.79%)	<0.001	103(32.1%)	106(33.0%)	0.959
	≧2 and<5	238(54.21%)	431(56.86%)		201(62.6%)	199(62.0%)	
	≧5	18(4.1%)	177(23.35%)		17(5.3%)	16(5.0%)	
**Nodal status**	N0	386(87.93%)	563(74.27%)	<0.001	275(85.7%)	270(84.1%)	0.509
	N1	46(10.48%)	129(17.02%)		41(12.8%)	41(12.8%)	
	N2	4(0.91%)	40(5.28%)		3(0.9%)	8(2.5%)	
	N3	3(0.68%)	26(3.43%)		2(0.6%)	2(0.6%)	
**ER**	Negative	349(79.5%)	622(82.06%)	0.276	264(82.2%)	262(81.6%)	0.837
	Positive	90(20.5%)	136(17.94%)		57(17.8%)	59(18.4%)	
**PR**	Negative	364(82.92%)	661(87.2%)	0.042	280(87.2%)	282(87.9%)	0.811
	Positive	75(17.08%)	97(12.8%)		41(12.8%)	39(12.1%)	
**Chemotherapy**	yes	334(76.08%)	529(69.79%)	0.019	247(76.9%)	251(78.2%)	0.705
	no	105(23.92%)	229(30.21%)		74(24.1%)	70(21.8%)	
**Radiotherapy**	yes	439(100%)	232(30.61%)		311(100%)	73(23.5%)	
	no	0(0%)	526(69.39%)		0(0%)	238(76.5%)	

^a^ PSM = propensity score matching.

^b^ BCS+ RT = Breast conserving surgery plus radiotherapy.

### Comparison of BCSS and OS between BCS+RT and mastectomy groups

**Fig 1:**
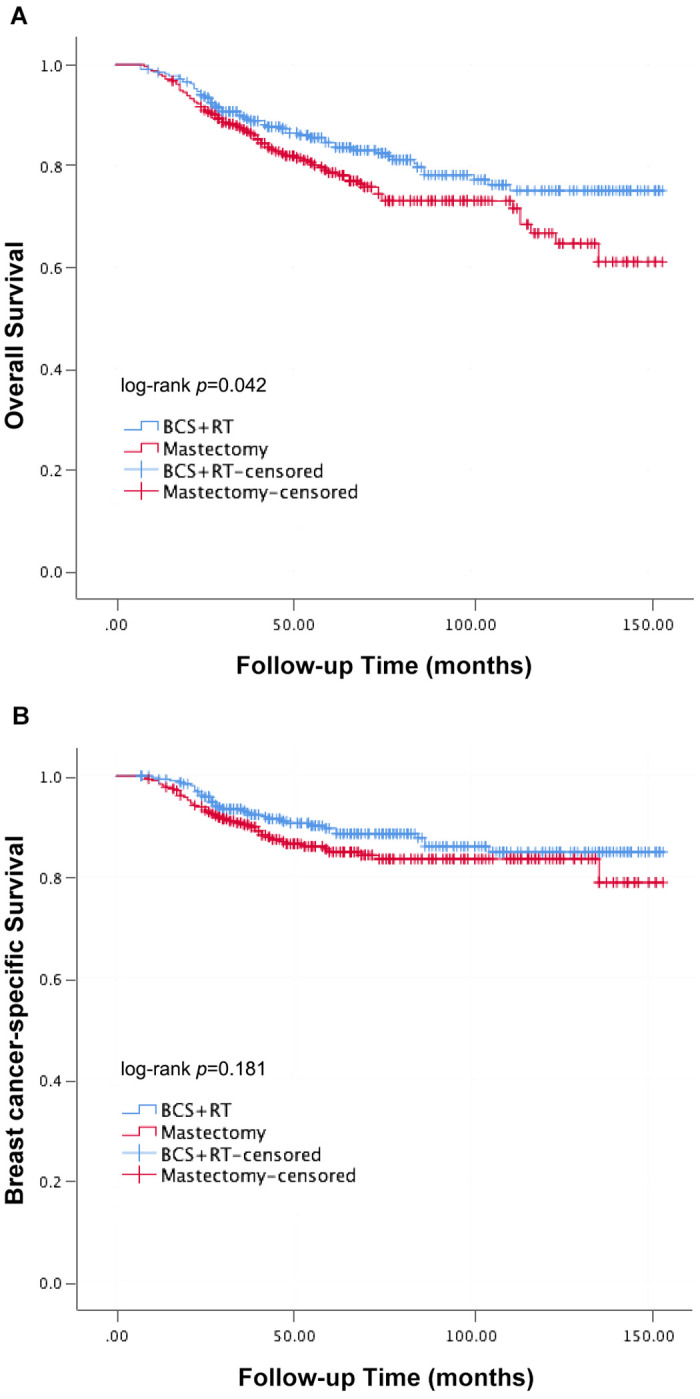
Kaplan-Meier curves of OS (A) and BCSS (B) between BCS+RT and mastectomy groups.

After 56 months median follow-up time, patients in the BCS + RT group showed a significantly higher OS than patients in the mastectomy group (log-rank *P* = 0.042, Fig 1A), while patients in the two subjects had similar BCSS (log-rank *P* = 0.181, Fig 1B). The 5-year OS for the group with BCS + RT was 84.6% and was 78.7% in the mastectomy group, while the 10-year OS for patients in the two groups was 75.1% and 66.7%. Similar 5-year and 10-year BCSS were found for the two groups (5-year:BCS + RT, 89.6% vs. mastectomy, 85.0%; 10-year:BCS + RT, 85.0% vs. mastectomy, 83.6%).

### Prognostic factors associated with OS and BCSS

After adjusting for the important prognostic variables in the univariate analysis (S1 Table), the results of multivariate cox regression analysis showed that patients who had larger tumors and more lymph node metastases showed poor BCSS and OS. Patients in the BCS + RT group showed a significantly higher OS outcomes compared to patients in the mastectomy group (HR = 0.684,95% CI = 0.479–0.977, *P* = 0.037). Patients who were not administered with chemotherapy showed lower OS compared to those who had received chemotherapy (HR = 2.253, 95% CI = 1.457–3.485, *P*<0.001) (Table 2).

**Table 2:** Prognostic factors for OS and BCSS in multivariate analysis.

Characteristics		OS^a^	*P*	BCSS^b^	*P*
		Multivariate		Multivariate	
**Year of diagnosis**	2004–2009	Ref.	Ref.	Ref.	Ref.
	2010–2014	0.989(0.675–1.450)	0.955	1.029(0.637–1.661)	0.908
**Age (years)**	20–49	Ref.	Ref.	Ref.	Ref.
	50–80	1.514(0.962–2.383)	0.073	1.175(0.688–2.005)	0.555
**Race**	White	Ref.	Ref.	Ref.	Ref.
	Black	1.211(0.720–2.036)	0.470	1.127(0.527–2.223)	0.729
	Other	0.984(0.470–2.058)	0.966	0.827(0.295–2.317)	0.718
**Marital status**	Married	Ref.	Ref.	Ref.	Ref.
	Not married	0.888(0.604–1.305)	0.546	0.883(0.537–1.450)	0.623
**Grade**	I	Ref.	Ref.	Ref.	Ref.
	II	1.054(0.230–4.821)	0.946	0.963(0.114–8.111)	0.972
	III	1.144(0.269–4.854)	0.856	0.875(0.115–6.687)	0.898
	IV	2.066(0.427–9.992)	0.367	1.722(0.191–15.555)	0.629
**Tumor size (cm)**	<2	Ref.	Ref.	Ref.	Ref.
	≧2 and<5	2.266(1.436–3.576)	<0.001	3.554(1.760–7.177)	<0.001
	≧5	5.593(2.682–11.664)	<0.001	7.159(2.668–19.209)	<0.001
**Nodal status**	N0	Ref.	Ref.	Ref.	Ref.
	N1	1.350(0.803–2.269)	0.257	1.752(0.973–3.155)	0.062
	N2	2.637(0.904–7.697)	0.076	4.276(1.386–13.187)	0.011
	N3	16.437(5.575–48.459)	<0.001	20.504(5.729–73.391)	<0.001
**ER**	Positive	Ref.	Ref.	Ref.	Ref.
	Negative	1.043(0.615–1.771)	0.875	1.205(0.596–2.436)	0.604
**PR**	Positive	Ref.	Ref.	Ref.	Ref.
	Negative	1.061(0.558–2.019)	0.856	0.908(0.407–2.027)	0.814
**Chemotherapy**	yes	Ref.	Ref.	Ref.	Ref.
	no	2.253(1.457–3.485)	<0.001	1.318(0.688–2.525)	0.405
**Surgical method**	BCS+RT	0.684(0.479–0.977)	0.037	0.741(0.468–1.173)	0.200
	Mastectomy	Ref.	Ref.	Ref.	Ref.

^a^ OS = overall survival.

^b^ BCSS = breast cancer-specific survival.

### Subgroup analysis of OS and BCSS

**Fig 2:**
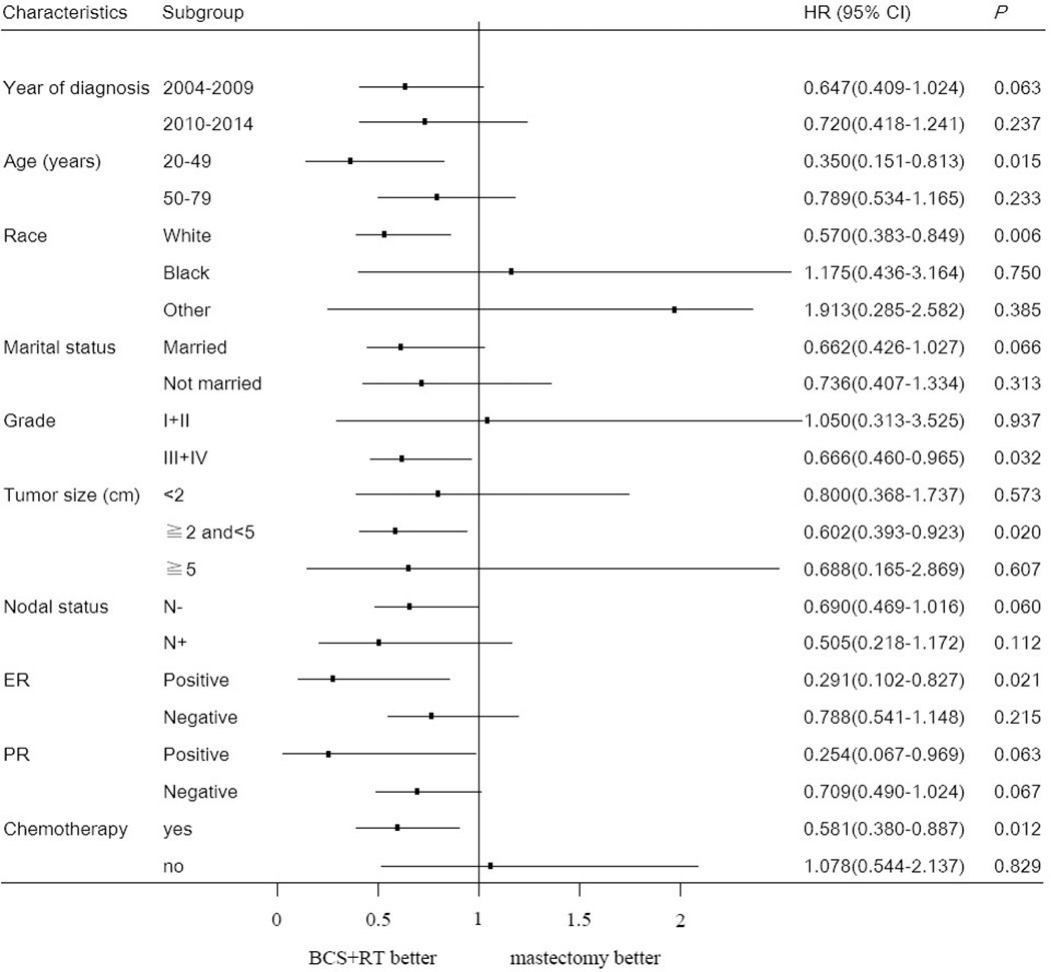
The forest plot for HR comparing OS between the BCS+RT group and mastectomy group according to different variables.

A subgroup analysis was performed to determine the possible factors affecting the survival time for patients who had been subjected to the two types of surgical procedures (Table 3). The subgroup analysis was based on age, diagnostic year, race, marital status, histological grade, tumor size (T stage), lymph node status, ER, PR, surgical methods, and postoperative chemotherapy. After adjusting for the important prognostic variables in the univariate analysis (S2 Table), the multivariate analysis showed significantly high OS when BCS+RT was given to patients aged between 20–49 years, the white race group, patients with grade III+IV, patients with T2, patients with ER positive, and those who received chemotherapy (S1 Fig). The subgroup analysis of OS is shown in Fig 2. No factors were associated with the BCSS of patients who received BCS+RT.

**Table 3:** Subgroup analysis of OS and BCSS in multivariate analysis.

Characteristics		OS^a^	*P*	BCSS^b^	*P*
		Multivariate		Multivariate	
**Year of diagnosis**	2004–2009	0.647(0.409–1.024)	0.063	0.928(0.506–1.700)	0.808
	2010–2014	0.720(0.418–1.241)	0.237	0.533(0.268–1.062)	0.074
**Age (years)**	20–49	0.350(0.151–0.813)	0.015	0.459(0.181–1.163)	0.101
	50–79	0.789(0.534–1.165)	0.233	0.847(0.502–1.429)	0.533
**Race**	White	0.570(0.383–0.849)	0.006	0.595(0.358–0.987)	0.054
	Black	1.175(0.436–3.164)	0.750	1.797(0.422–7.641)	0.428
	Other	1.913(0.285–2.582)	0.385	21.271(0.000–3.688)	0.964
**Marital status**	Married	0.662(0.426–1.027)	0.066	0.698(0.397–1.227)	0.211
	Not married	0.736(0.407–1.334)	0.313	0.713(0.334–1.526)	0.384
**Grade**	I+II	1.050(0.313–3.525)	0.937	0.361(0.058–2.242)	0.274
	III+IV	0.666(0.460–0.965)	0.032	0.738(0.461–1.182)	0.206
**Tumor size (cm)**	<2	0.800(0.368–1.737)	0.573	0.451(0.111–1.839)	0.267
	≧2 and<5	0.602(0.393–0.923)	0.020	0.757(0.452–1.266)	0.288
	≧5	0.688(0.165–2.869)	0.607	0.850(0.152–4.735)	0.852
**Nodal status**	N-	0.690(0.469–1.016)	0.060	0.722(0.428–1.220)	0.224
	N+	0.505(0.218–1.172)	0.112	0.683(0.284–1.647)	0.396
**ER**	Positive	0.291(0.102–0.827)	0.021	0.284(0.073–1.108)	0.070
	Negative	0.788(0.541–1.148)	0.215	0.829(0.511–1.344)	0.446
**PR**	Positive	0.254(0.067–0.969)	0.063	0.173(0.032–0.921)	0.055
	Negative	0.709(0.490–1.024)	0.067	0.800(0.497–1.288)	0.359
**Chemotherapy**	yes	0.581(0.380–0.887)	0.012	0.751(0.458–1.230)	0.255
	no	1.078(0.544–2.137)	0.829	0.538(0.166–1.749)	0.303

^a^ OS = overall survival.

^b^ BCSS = breast cancer-specific survival.

## Discussion

The prognosis of MBC patients after being subjected to BCS + RT and mastectomy has not been established. We found that patients subjected to BCS + RT exhibited better OS outcomes when compared to patients who had been subjected to mastectomy. Furthermore, patients who had been subjected to BCS+RT exhibited improved OS outcomes in the young, white race, grade III+IV, T2, ER positive, and chemotherapy subgroups. The OS and BCSS outcomes of mastectomy were not better than those of BCS + RT in any subgroup.

In our study, before PSM, 80.87% of the MBC cases were classified as grade III. Most tumors are larger than 2 cm (72.18%), and most of the cases were negative for ER and PR (81.12%, 85.63%). These findings indicate a poor prognosis of MBC, consistent with the study findings of Chao et al. [19]. A low positive rate of axillary lymph nodes was observed in our study. 74.27% of the patients in the mastectomy group and 87.93% of the patients in the BCS + RT group had no lymph node metastases, which is in tandem with previous findings (20.72%) [20, 21]. Compared to lymphatic metastasis, MBC is highly associated with blood metastasis; therefore, the rate of lymph node metastasis in MBC patients is low [5].

Like other studies, this study established that tumor grade and hormone receptor status was not correlated with MBC prognosis while the tumor size and lymph node metastasis were associated with it [22, 23]. In our study, patients who were not administered with chemotherapy showed lower OS than those who had received chemotherapy (HR = 2.253, 95% CI = 1.457–3.485, *P*<0.001). At present, whether chemotherapy is beneficial to the prognosis of MBC is still controversial. A previous study documented that the OS outcomes of patients administered with chemotherapy were better than the OS of patients who had not been administered with chemotherapy [24]. Studies have also documented that chemotherapy does not improve the OS of MBC patients because MBC is resistant to chemotherapy [25, 26]. We think that whether chemotherapy is effective for MBC or not should be verified by further study.

In our study, the OS outcomes for the BCS + RT group were significantly higher when compared to that of the mastectomy group. However, the BCSS outcomes of the two groups were statistically comparable. Zhang et al. [27] also compared the overall OS and BCSS of the BCS+RT and mastectomy groups in metaplastic breast cancer using the SEER database. Different from our results, they conclude that both the OS and BCSS of the BCS + RT group are better than that of mastectomy group. The difference between the two results may be that Zhang et al. directly reached a conclusion through regression analysis without performing PSM on the two different groups. The same as our research, BCS + RT and mastectomy groups in Zhang et al. are clearly different. The patients in BCS + RT group presented with smaller tumors and less lymph node metastases. Despite a higher stage of disease, less patients in the mastectomy group received chemotherapy. Without PSM, the better prognosis in the BCS + RT group may be due to the earlier disease stage of the BCS + RT group rather than the surgical method. Although using PSM result in a smaller sample size which can lead to reduced statistical power. That may be the reason why BCSS was not statistically significant in our study. Dave et al. [15] reported that the BCS and the mastectomy group had statistically similar 5-year local recurrence-free rate (88% vs. 85%, P = 0.86), disease-free rate (55% vs. 84%, P = 0.13), and overall survival rate (80% vs. 89%, P = 0.58). The reason why their results are different from ours may be attributed to the fact that all BCS patients enrolled in our study were subjected to postoperative radiotherapy, but the postoperative radiotherapy rate for BCS patients in the study of Dave et al. was 86.36%. Postoperative radiotherapy in BCS patients inhibits local recurrence and improves the overall survival rate [8, 28]. Li et al. [29] concluded that MBC patients could benefit from radiotherapy through SEER database analysis. Wang et al. [30] also confirmed that PMRT could improve the BCSS of MBC patients with intermediate-and high-risk disease. In addition, a recent study showed that postoperative breast-conserving radiotherapy significantly improved the OS outcomes for MBC patients compared to the OS outcomes for MBC patients who had not been administered with radiotherapy (5-year OS: 85% vs. 61%, 10-year OS: 67% vs. 49%, P <0.001) [20]. Only 23.5% of our patients in the mastectomy group received radiotherapy, so we think that the better prognosis of the BCS + RT group than that of the mastectomy group may be attributed to the high radiotherapy rate of the BCS + RT group.

The difference in results between OS and BCSS may require further explanation. It means that the OS of the mastectomy group is lower due to causes other than breast cancer. Older patients are thought to more often receive mastectomy than BCS + RT. These patients have lower survival because of age [13]. Some of the women underwent mastectomy due to an overall judgment of their health situation. Patients with poor health and more complications are more likely to choose mastectomy [31]. More complications also lead to an increase in non-breast cancer mortality. In the United States, where women with a higher socioeconomic status are more likely to undergo BCT [32, 33]. Higher socioeconomic status means better medical conditions, which means lower mortality rates for non-breast cancer. But there may not be a necessary connection between the two. BCS+RT provides better health-related quality of life and was associated with fewer postoperative complications than mastectomy. This may be related to lower non-breast cancer mortality in BCS + RT.

Our study has some limitations. Firstly, despite the use of propensity matched landmark analysis, there may be residual confounding factors. Secondly, since the SEER database has only recorded the status of HER2 since 2010, we did not collect the status of HER2. Thirdly, the SEER database did not provide details of the irradiated technique and scope, lack of local regional recurrence data, and has no records on Ki-67, endocrine therapy and targeted therapy. Finally, as metaplastic breast cancer is a rare type of breast cancer, the number of 321 patients in each group is not small, but it may have weak power in statistical analysis. Despite these limitations, our research is still very meaningful. It provides a certain theoretical basis for the choice of surgical methods for metaplastic cancer.

## Conclusion

In conclusion, this population-based study based on the SEER database showed that the OS of MBC patients receiving BCS + RT was significantly better than that of patients receiving mastectomy. Therefore, BCS + RT may be the preferable choice for MBC patients, but the comprehensive factors such as patient’s health status, economic level and patient’s willingness should also be considered.

## Supporting information

S1 Table Prognostic factors for OS and BCSS in univariate analysis. (DOCX)

S2 Table Subgroup analysis of OS and BCSS in univariate analysis. (DOCX)

S1 Fig OS of MBC patients displayed as Kaplan–Meier curves according to surgical method for different patient subgroups. (JPG)
