# Supplementary material for: The different outcomes between breast-conserving surgery plus radiotherapy and mastectomy in metaplastic breast cancer: A population-based study
Source: PLoS One. 2021 Sep 2;16(9):e0256893. doi: 10.1371/journal.pone.0256893 (PMC8412345; doi:10.1371/journal.pone.0256893)
Supplement: S1 Table — (DOCX) [file pone.0256893.s001.docx]

S1 Table. Prognostic factors for OS and BCSS in univariate analysis

| Characteristics |  | OS |  | BCSS |  |
| --- | --- | --- | --- | --- | --- |
|  |  | Univariate | *P* | Univariate | *P* |
| Year of diagnosis | 2004-2009 | Ref. | Ref. | Ref. | Ref. |
|  | 2010-2014 | 0.898(0.619-1.302) | 0.571 | 0.870(0.548-1.381) | 0.554 |
| Age (years) | 20-49 | Ref. | Ref. | Ref. | Ref. |
|  | 50-80 | 1.385(0.907-2.117) | 0.132 | 0.956(0.580-1.576) | 0.860 |
| Race | White | Ref. | Ref. | Ref. | Ref. |
|  | Black | 1.135(0.696-1.851) | 0.613 | 1.065(.562-2.020) | 0.846 |
|  | Other | 1.048(0.510-2.153) | 0.898 | 0.886(0.323-2.435) | 0.815 |
| Marital status | Married | Ref. | Ref. | Ref. | Ref. |
|  | Not married | 1.112(0.778-1.590) | 0.560 | 1.063(0.670-1.686) | 0.795 |
| Grade | I | Ref. | Ref. | Ref. | Ref. |
|  | II | 1.294(0.292-5.735) | 0.734 | 1.398(0.172-11.366) | 0.754 |
|  | III | 1.218(0.301-4.935) | 0.782 | 1.518(0.211-10.938) | 0.679 |
|  | IV | 2.472(0.534-11.448) | 0.247 | 2.845(0.322-24.361) | 0.340 |
| Tumor size (cm) | <2 | Ref. | Ref. | Ref. | Ref. |
|  | ≧2 and<5 | 1.741(1.139-2.661) | **0.010** | 3.272(1.675-6.391) | **0.001** |
|  | ≧5 | 3.778(1.956-7.296) | **<0.001** | 7.486(3.041-18.431) | **<0.001** |
| Nodal status | N0 | Ref. | Ref. | Ref. | Ref. |
|  | N1 | 1.227(0.744-2.023) | 0.423 | 1.870(1.059-3.304) | **0.031** |
|  | N2 | 2.476(0.912-6.723) | 0.075 | 4.482(1.626-12.356) | **0.004** |
|  | N3 | 16.380(5.869-45.714) | **<0.001** | 18.923(5.754-62.230) | **<0.001** |
| ER | Positive | Ref. | Ref. | Ref. | Ref. |
|  | Negative | 1.088(0.675-1.752) | 0.730 | 1.140(0.617-2.110) | 0.675 |
| PR | Positive | Ref. | Ref. | Ref. | Ref. |
|  | Negative | 1.260(0.710-2.234) | 0.430 | 1.096(0.547-2.194) | 0.796 |
| Chemotherapy | yes | Ref. | Ref. | Ref. | Ref. |
|  | no | 1.573(1.082-2.289) | **0.018** | 0.786(0.441-1.400) | 0.413 |
| Surgical method | BCS+RT | 0.701( 0.496-0.990) | **0.044** | 0.739( 0.474-1.153) | 0.183 |
|  | Mastectomy | Ref. | Ref. | Ref. | Ref. |

OS= overall survival; BCSS = breast cancer-specific survival
