# Supplementary material for: The different outcomes between breast-conserving surgery plus radiotherapy and mastectomy in metaplastic breast cancer: A population-based study
Source: PLoS One. 2021 Sep 2;16(9):e0256893. doi: 10.1371/journal.pone.0256893 (PMC8412345; doi:10.1371/journal.pone.0256893)
Supplement: S2 Table — (DOCX) [file pone.0256893.s002.docx]

S2 Table. Subgroup analysis of OS and BCSS in univariate analysis

| Characteristics |  | OS |  | BCSS |  |
| --- | --- | --- | --- | --- | --- |
|  |  | Univariate | *P* | Univariate | *P* |
| Year of diagnosis | 2004-2009 | 0.667(0.423-1.051) | 0.081 | 0.932(0.512-1.697) | 0.819 |
|  | 2010-2014 | 0.747(0.438-1.274) | 0.284 | 0.549(0.278-1.085) | 0.084 |
| Age (years) | 20-49 | 0.360(0.158-0.823) | **0.015** | 0.429(0.173-1.063) | 0.068 |
|  | 50-79 | 0.830(0.565-1.221) | 0.345 | 0.890(0.532-1.491) | 0.659 |
| Race | White | 0.594(0.400-0.880) | **0.009** | 0.595(0.360-0.983) | **0.043** |
|  | Black | 1.362(0.536-3.460) | 0.517 | 2.168(0.575-8.176) | 0.253 |
|  | Other | 1.178(0.290-4.788) | 0.819 | 1.441(0.203-10.239) | 0.715 |
| Marital status | Married | 0.718(0.466-1.105) | 0.132 | 0.765(0.440-1.329) | 0.341 |
|  | Not married | 0.678(0.380-1.209) | 0.187 | 0.694(0.328-1.468) | 0.339 |
| Grade | I+II | 0.958(0.346-2.650) | 0.934 | 0.655(0.156-2.751) | 0.564 |
|  | III+IV | 0.689(0.477-0.995) | **0.047** | 0.760(0.476-1.214) | 0.251 |
| Tumor size (cm) | <2 | 0.780(0.370-1.642) | 0.513 | 0.408(0.105-1.583) | 0.195 |
|  | ≧2 and<5 | 0.641(0.421-0.976) | 0.038 | 0.762(0.458-1.268) | 0.295 |
|  | ≧5 | 0.755(0.252-2.257) | 0.615 | 0.843(0.225-3.154) | 0.800 |
| Nodal status | N- | 0.712(0.484-1.048) | 0.085 | 0.759(0.450-1.280) | 0.302 |
|  | N+ | 0.650(0.295-1.433) | 0.285 | 0.718(0.307-1.679) | 0.444 |
| ER | Positive | 0.328(0.119-0.903) | 0.031 | 0.334(0.090-1.233) | 0.100 |
|  | Negative | 0.797(0.549-1.158) | 0.235 | 0.842(0.521-1.360) | 0.482 |
| PR | Positive | 0.357(0.109-1.165) | 0.088 | 0.240(0.050-1.159) | 0.076 |
|  | Negative | 0.750(0.522-1.079) | 0.122 | 0.838(0.524-1.341) | 0.461 |
| Chemotherapy | yes | 0.598(0.393-0.909) | 0.016 | 0.765(0.469-1.274) | 0.282 |
|  | no | 0.986(0.525-1.854) | 0.966 | 0.646(0.223-1.868) | 0.420 |

OS= overall survival; BCSS = breast cancer-specific survival
