# Supplementary figures and images for: The different outcomes between breast-conserving surgery plus radiotherapy and mastectomy in metaplastic breast cancer: A population-based study
Source: PLoS One. 2021 Sep 2;16(9):e0256893. doi: 10.1371/journal.pone.0256893 (PMC8412345; doi:10.1371/journal.pone.0256893)

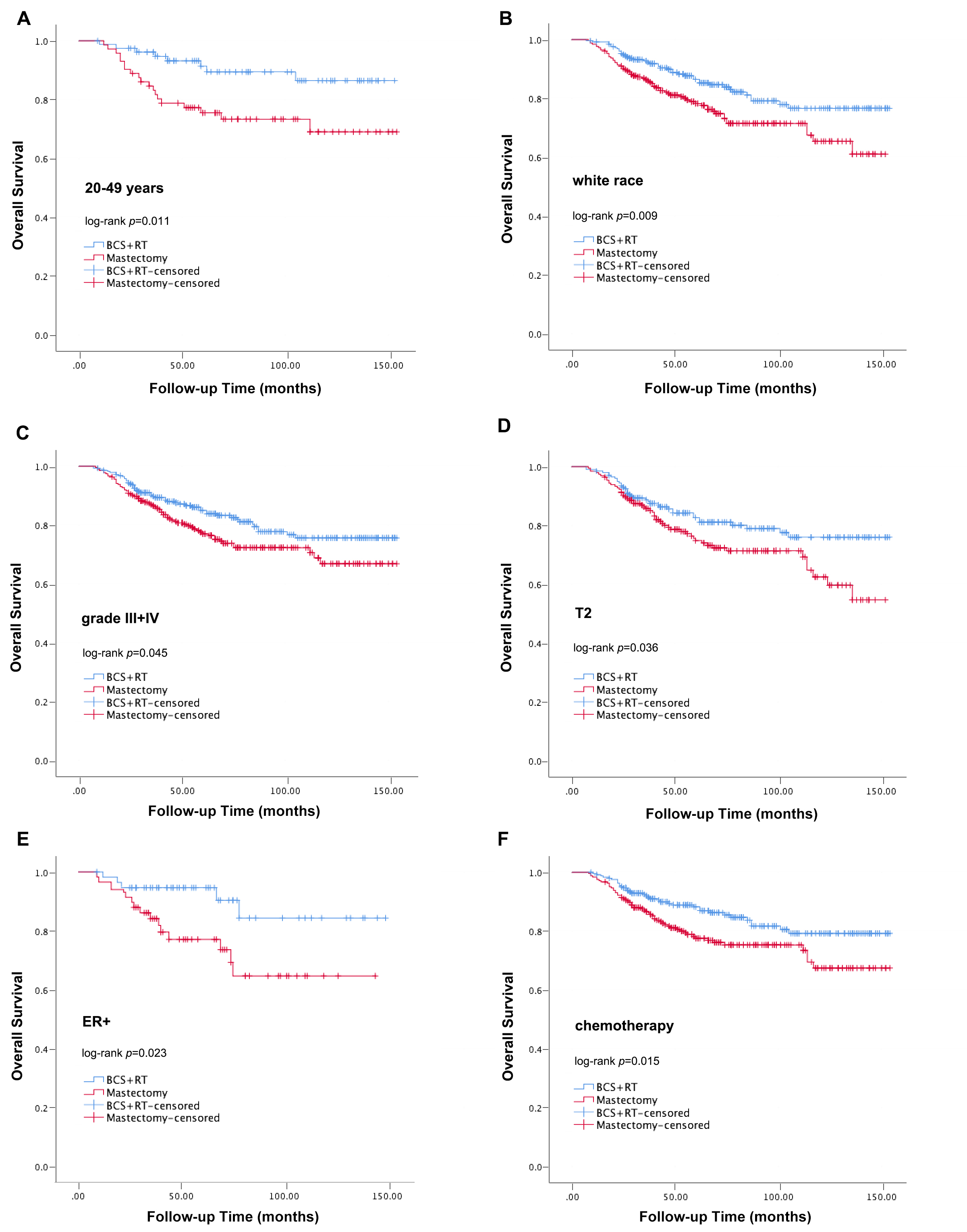

Supplement: S1 Fig — (JPG) [file pone.0256893.s003.jpg]
